# Regulatory effect of Garidisan on dysbiosis of the gut microbiota in the mouse model of ulcerative colitis induced by dextran sulfate sodium

**DOI:** 10.1186/s12906-019-2750-y

**Published:** 2019-11-21

**Authors:** Ling-yan Pei, Yu-shi Ke, Huan-hu Zhao, Wei-zhi Liu, Lin Wang, Chao Jia, Meng-ni Shi, Qian-hui Fu, Jian Cui, Shu-chun Li

**Affiliations:** 1 School of Pharmacy, Minzu University of China, 27 South Street, Zhongguancun, Beijing, 100081 China; 2 Ministry of Education, Key Laboratory of Ethnomedicine (Minzu University of China), Beijing, 100081 China; 3 Center for Drug Evaluation, China Food and Drug Administration, Beijing, 100081 China; 4 Department of Histology and Embryology, Xinxiang Medical University, Xinxiang, 453003 Henan China; 5 Department of Pathology, National Center for Children’s Health, Beijing Children’s Hospital, Capital Medical University, Beijing, 100045 China

**Keywords:** Ulcerative colitis, Gut microbiota, Microbiology

## Abstract

**Background:**

Ulcerative colitis (UC) is a modern refractory disease, and its etiology has been difficult to discern. Studies have shown that UC is closely associated with the gut microbiota. Garidisan is composed of wild poppy and *Artemisia frigida* Willd and is commonly used for the treatment of UC in Inner Mongolia, China. In clinical settings, Garidisan has been found to treat UC effectively, with low recurrence. Previous studies have shown that Garidisan has a good therapeutic effect on mice with UC, but the therapeutic mechanism is still unclear. In this study, we investigated the regulatory effect of Garidisan on dysbiosis of the gut microbiota in a UC mouse model and explored the possible mechanism of the therapeutic effect of Garidisan on UC.

**Methods:**

The UC mouse model was established by the dextran sulfate sodium (DSS) circulating free water drinking method, and the luminal contents were sampled under sterile conditions. High-throughput sequencing of the 16S rRNA gene V3 + V4 region of the luminal contents of the control group, model group, and Garidisan group was conducted, and clustering of operational taxonomic units (OTUs) and species annotation were performed. The differences in species composition and microbial community structure between individual groups of samples were analyzed using MetaStat, LefSe, rank sum test, and Bayesian causal network analysis.

**Results:**

The UC mouse model was successfully established and the sequencing results were of adequate quality. There were significant differences in the diversity of luminal contents between the control group, model group, and Garidisan group, and the differences between groups were greater than those within any group. The therapeutic effect of Garidisan on UC is attributed to the direct effect on the *Lachnospiraceae* family of bacteria.

**Conclusion:**

Garidisan has a good regulatory effect on the gut microbiota, and *Lachnospiraceae* could be an important direct target of Garidisan for the treatment of UC.

## Background

Ulcerative colitis (UC) is a refractory, spontaneous inflammatory bowel disease involving the rectum and colonic mucosa. UC has a wide range of lesions, high recurrence, poor therapeutic effects, and high possibility of carcinogenesis. It has been listed as a modern refractory disease by the World Health Organization. Studies conducted during the past decade have shown that environmental factors, particularly the gut microbiota, and genetic and immune factors, may be the main pathological factors in the pathogenesis of UC [1]. Histopathological changes associated with UC are characterized by diffusive tissue inflammatory responses, including ulceration, crypt abscesses, small vessel inflammation, goblet cell reduction, and non-specific manifestations of various types of inflammatory cell infiltration.

Because pathogenesis of UC has not been fully determined, conventional treatment methods for this disease currently include amino salicylic acid, hormones, immunosuppressive agents, biological agents, andtraditional herbal medicine. Sulfasalazine is the most widely used drug for the treatment of UC, especially that of mild to moderate UC. Sulfasalazine (salazosulfapyridine, SASP) is a prodrug composed by a molecule of 5-aminosalicylic acid (5-ASA) and sulfapyridine, linked by an azo bond. It is suitable for most patients to maintain sustained treatment. Glucocorticoids are the first choice for the treatment of severe and fulminant UC. Immunosuppressive agents are mainly used in patients in whom salicylic acid or glucocorticoid therapy has failed, and those suffering from glucocorticoid toxicity or long-term dependence on glucocorticoids. Hormone therapy like corticosteroids, biological agents such as anti-tumor necrosis factor (TNF-α) monoclonal antibodies and traditional herbal medicine have been used for the treatment of UC. The treatment of UC using Western medicine is usually associated with significant adverse effects. UC is likely to relapse after treatment, and it is highly difficult to cure. Although the therapeutic effect of traditional herbal medicine is good, patient compliance is usually challenging. Therefore, there is currently an urgent need to explore new entry points for the treatment of UC.

Plants and natural products have a long history of use in the treatment of various diseases. They tend to feature low cost, multi-targeting, and low rates of toxic side effects. Garidisan is a drug commonly used for the treatment of UC in Inner Mongolia, China. It consists of wild poppy and *Artemisia frigida* Willd, and years of clinical applications have proven that it can effectively treat UC without toxic side effects. UC treated by Garidisan is unlikely to relapse after healing, and patients enjoy rapid restoration of bowel symptom [2]. However, the pharmacological mechanism underlying the use of Garidisan for the treatment of UC is unclear. In recent years, studies have demonstrated that dysbiosis of the gut microbiota is closely related to the incidence of UC [3, 4]. There was a clear increase in *Bacteroides* and *Clostridium* in the luminal contents of DSS-induced UC mice. Specifically, *Bacteroides distasonis* and *Clostridium ramosum* were significantly increased [5]. The increase in the copy number of 16S rRNA gene in *Akkermansia muciniphila* and *Enterobacter* was related to the disease activity of DSS-induced mice. *Bifidobacteria* and *Lactobacillus* were elevated in inflammatory bowel diseases, while butyric acid-producing bacteria were missing [6]. In our previous study, Garidisan can effectively repair the damaged mucosa of TNBS-induced UC model rat, and its efficacy is comparable to that of sulfasalazine [7]. Garidisan can reduce the content of TGF-β, IL-6 and IL-17, increase the content of IFN-γ, regulate the differentiation direction of TH0 cells in UC model rats and restore the balance of Th1/Th17 to achieve the purpose of treating UC [8]. Our further studies found that Garidisan reduces the infiltration of inflammatory cells by regulating the balance of immune cells, pro-inflammatory factors, and anti-inflammatory factors and by reducing the expression of ICAM-1, thereby promoting the maturation of regenerated tissues. Furthermore, Garidisan has also been found to promote the functional maturity of regenerative tissues by reducing collagen formation, promoting the maturation of new blood vessels, and increasing the expression of growth factors and their receptors [9]. Here, we explored the dysbiosis of the gut microbiota and its targeting biomarkers in UC model mice using high-throughput sequencing and bioinformatics analysis to further explore the mechanism of Garidisan in UC treatment.

## Methods

### Chemical

70% Ethanol, Dextran sulfate sodium (M.W. 36,000–50,000, American MP), Fecal genomic DNA extraction kit (Tiangen Biochemical Technology Co., Ltd., DP304), Sulfasalazine (Sine [Tianping] Pharmaceutical Co., Ltd., Shanghai, China), Bupiyichangwan (Guangzhou Chen Li Ji Pharmaceu- tical Factory Co., Ltd., Guangdong Province, China) et al.

### Experimental animals

A total of 50 male C57BALB/c mice, SPF grade, body weight of 22 g ± 2 g, 9 weeks old, were provided by Beijing Weitong Lihua Experimental Animal Technology Co., Ltd. under license number: SCXK (Beijing) 2012–0001. Mice with 3–5 per cage were housed in the SPF laboratory animal facility (SYKX (Beijing) 2016–0010) of the Science and Technology Research Institute of the National Health and Family Planning Commission. All mice were maintained at 24 ± 1 °C, 55 ± 1% humidity with a 14:10-h light–dark cycle. Food and water were provided ad libitum. All experimental measures were carried out in accordance with the approved guidelines (Guidelines for the Care and Use of Laboratory Animals) established by the Chinese Council on Animal Care.

### Composition and preparation of Mongolian medicine—Garidian

The Mongolian medical drug Garidisan consists of 15 g *Papaver nudicaule* L. and 24 g *Artemisia frigida* Willd. We purchased both from Inner Mongolia Herbal Medicine Purchasing and Supply Company. The wild poppy and *Artemisia frigida* Willd were weighed out in the correct ratio, and then 22 times volume of 70% ethanol was added and the mixture was allowed to reflux for 5 h [9]. The extract was concentrated to 0.59 g/mL containing crude medicine and stored at 4 °C in the dark.

### Replicate the ulcerative colitis mouse model and administrate the drug

Among various chemically induced colitis models, DSS-induced colitis model is widely used because of its simplicity and many similarities with human ulcerative colitis. DSS carries a highly negative charge contributed by sulfate groups, is toxic to the colonic epithelia, and induces erosions that ultimately compromise barrier integrity resulting in increased colonic epithelial permeability [5, 10, 11]. The UC mouse model was established according to the method described by Benoit Chassaing et al. [10], and mice in the control group were given sterile drinking water. The UC mouse model establishment was divided into 3 cycles: During the first cycle, mice were given free access to 2.5% w/v DSS (M.W. 36000–50000, American MP) drinking solution. After 7 days, mice were given free access to normal sterile drinking water for the next 7 days. During the second cycle, mice were given free access to 2% w/v DSS drinking solution. After 7 days, they were given free access to normal sterile drinking water for an additional 7 days. During the third cycle, mice were given free access to 2.5% w/v DSS solution for 2 days, and then the concentration of DSS solution was lowered to 2%, w/v and mice were given free access to this 2% w/v DSS drinking solution for another 5 days, after which mice were given free access to normal sterile drinking water for an additional 2 days. After the UC model was established, the animals were sacrificed by cervical dislocation and colons were excised and prepared for histopathological examination. The experimental protocols were performed after approval and in accordance with the guidelines set by the Ethical Committee of Minzu University of China (Protocol number: 201702). Because Bupiyichang Pill is the most commonly used traditional herbal medicine for the treatment of UC, it has been marketed in China and is proven to be effective in improving UC. We set up the Bupiyichang Pill group as a traditional herbal medicine control group. Each group was given the corresponding drug during model establishment. The route of administration is intragastric administration. The model group means that the mice are randomly consumed with the DSS solution during the modeling process and sterile drinking water is administered during the administration. Since we used the intragastric administration method to give the corresponding doses of the drugs to each group of mice at the same time of model replication, in order to ensure the uniformity of the external conditions of each group of mice, we gave the same dose of sterile drinking water to the model group mice. The grouping and drug administration are described in Table 1. Day one for DAI recording was the day of the free access to DSS drinking solution. A mice disease activity index was recorded every day (Table 2) [9]. The disease index scores of seven values were added and divided into seven for statistical analysis. The DAI data were expressed as means±SEM. Statistical analysis was performed with Statistical Package for Social Science version 23.0 software (SPSS Inc., Chicago, IL). ANOVA was used for analysis.

**Table 1:** Grouping and medication

Group	Dosing
Control group	Sterile drinking water
Model group	DSS solution +Sterile drinking water
Sulfasalazine group	0.61 g/kg mouse body weight
Bupiyichang pill group	2.73 g/kg mouse body weight
Garidisan group	5.915 g/kg mouse body weight

**Table 2:** Scoring of disease activity index

Observation index	0	1	2
Weight change	Increase or Decrease< 5%	Increase or Decrease 5%≦10%	Increase or Decrease > 10%
Hair condition	Neat and shiny	Messy and shiny	Messy and matte
Activity	Normal	Less activity	Inactive
Fecal shape	well-formed	Loose stool	Liquid stool
Stool with mucus	None	Small amount	Mass
Stool with pus	None	Small amount	Mass
Stool with blood	none	a small amount visible to the naked eye	Visible obviously to the naked eye

### Isolating luminal contents of the colon

The colon tissue was longitudinally dissected and the contents were removed with sterile ophthalmological tweezers until no visible particles could be observed by the naked eye. The contents were placed in a 2-mL sterile cryotube and frozen in liquid nitrogen.

### Total DNA extraction

The total DNA of the luminal content was extracted using a mouse fecal genomic DNA extraction kit (Tiangen Biochemical Technology Co., Ltd., DP304).

### 16S rRNA sequence amplification and high-throughput sequencing

After genomic DNA extraction was completed, the purity and concentration of the extracted DNA were evaluated using agarose gel electrophoresis. An appropriate amount of the sample was diluted to 1 ng/μL with sterile water for PCR amplification. Primers used were as follows: 341F: CCTAYGGGRBGCASCAG; 806R: GGACTACNNGGGTATCTAAT. The PCR reaction system (30 μL) was as follows: Phusion Master Mix (2×), 15 μL; Primer (2 μM), 3 μL; gDNA (1 ng/μL), 10 μL (5–10 ng); H_2_O, 2 μL. The reaction procedure was as follows: pre-denaturation at 98 °C for 1 min; 30 cycles of 98 °C 10 s, 50 °C 30 s, and 72 °C 30 s; 72 °C 5 min. The PCR product was electrophoresed using a 2% agarose gel. The samples were mixed at the same concentration according to the concentration of the PCR product. After thorough mixing, the PCR product was purified by electrophoresis using a 2% agarose gel made with 1 × TAE. The products with the main band size between 400 and 450 bp were selected, and the target band was recovered. The purification kit selected was a Thermo Scientific GeneJET Gel Recovery Kit. The library was constructed using a NEB Next® UltraTM DNA Library Prep Kit for Illumina Library (New England Biolabs), and the library was subjected to Qubit quantification and library detection. The HiSeq sequencing platform was used for sequencing. A total of 6,130,487 effective sequences were obtained by sequencing. After removing contaminated, linker and invalid sequences, 4,907,035 sequences were obtained. For each sample Q20 > 98%, Q30 ≥ 97%.

### Bioinformatics and biostatistical analysis

After sequencing, the raw data were spliced and filtered to obtain clean data, operational taxonomic unit (OTU) clustering and species classification analysis were performed, and a representative sequence of each OTU was annotated according to the OTU clustering results. The corresponding species information and abundance distribution of species were obtained. Then, the OTUs were analyzed for abundance and alpha diversity calculation and a Venn diagram was drawn. The abundance and evenness of species, unique OTUs of each group, and OTUs shared by the groups were obtained. Using multi-sequence alignment of OTUs and the construction of a phylogenetic tree, differences in colony structures between different samples and groups were further obtained, and dimensionality reduction maps of Principal Co-ordinates analysis (PCoA), Principal component analysis (PCA), and non-metric multidimentional scaling (NMDS) were used for analysis. We used an improved statistical method for analysis of metagenomic data (MetaStat), linear discriminant analysis effect size (LEfSe), rank sum test, and Bayesian causal network analysis for statistical analysis of differences in species composition and microbial community structure between the groups.

## Results

### Animal model assessment

**Fig. 1:**
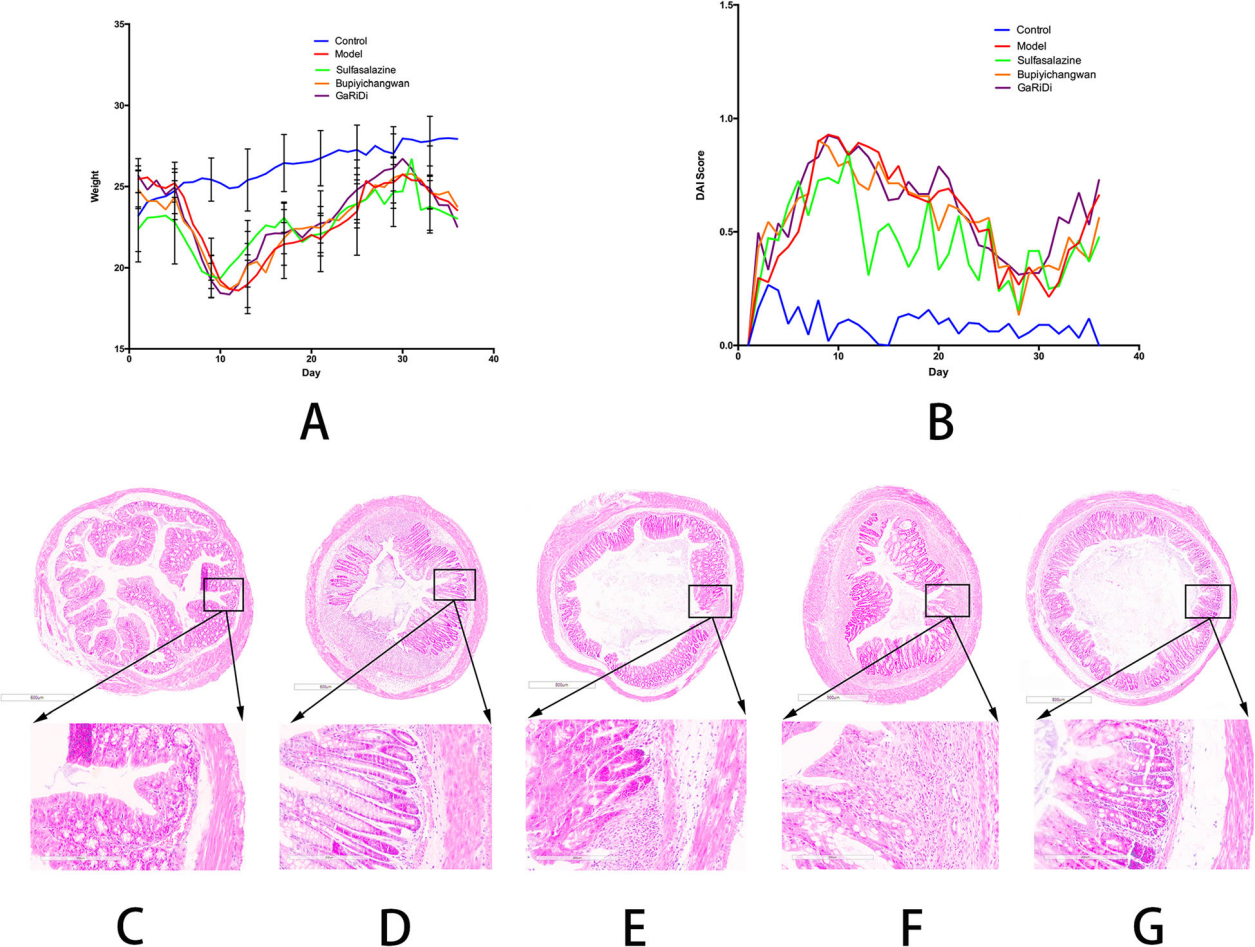
Evaluation of dextran sulfate sodium induced UC mouse model. **a** DAI score index; **b** Body weight change curve; **c** Pathological evaluation results of colon tissues in the control group. The upper image is 4.5×, and the lower image is 20×; **d** Pathological evaluation results of colon tissues in the model group. The upper image is 4.5×, and the lower image is 20×; **e** Pathological evaluation results of colon tissues in the sulfasalazine-treated group. The upper image is 4.5×, and the lower image is 20×; **f** Pathological evaluation results of colon tissues in the Bupiyichang pill-treated group. The upper image is 4.5×, and the lower image is 20×; **g** Pathological evaluation results of colon tissues in the Garidisan-treated group. The upper image is 4.5×, and the lower image is 20×

The body weight of mice was recorded every day during model establishment and drug administration. The body weight of the control group increased steadily. The body weight of the model group showed a rapid decline during the acute phase of model establishment and then a slow upward trend in the middle stage of model establishment, whereas the body weight remained lower than in the control group. The body weight of mice in the positive control group treated with sulfasalazine and Bupiyichang pills increased gradually after the acute phase. The body weight of mice in the Garidisan group was close to that of the positive control group. Especially, during the 20–30 days of drug administration, the body weight of mice in the Garidisan group was higher than in the positive control group (Fig. 1). During model establishment and drug administration, the disease activity index (DAI) of mice in each group was scored every day in addition to recording the changes in body weight, to dynamically monitored the establishment process. In this study, the UC mouse model was replicated and at the same time the corresponding drugs were administered to mice of different groups except the control group. Therefore, there was no significant difference in the DAI index between the model group and the other groups except the control group. Unlike the control group, the other four groups were in the acute phase of model establishment during the first week, and the DAI index showed an upward trend. In the middle stage of model establishment, the DAI index of the four groups decreased slightly, but it was still higher than in the control group. DSS was administered during the last week of model establishment. During that period, the DAI of each group showed a clear upward trend. The DAI index was lower in the sulfasalazine-treated group than in the control group and other drug-treated groups (Fig. 1).

After scanning pathological sections with the Leica APERIO AT2 high-throughput scanner, the pathological sections were observed under 4.5× and 20× magnifications. The four-layer structure of the colon wall was clear under low magnification. The model group showed obvious ulcers. The lamina propria and submucosa in the control group were thin, and the large intestine glands were regularly arranged. The tunica muscularis mucosa of the model group was readily visible. There were a large number of inflammatory cells in the lamina propria, especially at the ulcers, where the mucosal epithelium was incomplete and the gut gland disappeared. In the Garidisan group, the mucosal epithelium was intact and the large intestine glands were regularly arranged. In the sulfasalazine and Bupiyichang pill groups, the mucosal epithelium was intact, whereas the large intestine glands were missing at the ulcer-healing area, and there were abundant inflammatory cells in the mucosal lamina propria. There were abundant inflammatory cells in the submucosa, especially in the Bupiyichang pill group. Under high magnification, the mucosal layers were intact. In the control group, the large intestine glands were regularly arranged. In the model group, there were obvious ulcers. The epithelium was incomplete and the large intestine glands were not visible at the ulcers. The tunica muscularis mucosa was intact. There were many inflammatory cells the in the lamina propria and submucosa sites. In the Garidisan group, the mucosal epithelium was intact. There were a limited number of inflammatory cells in the lamina propria, and the tunica muscularis mucosa was intact. In the sulfasalazine-treated group, the mucosal epithelium was intact at the ulcer-healing sites, and there were many inflammatory cells in the lamina propria, and few glands. In the Bupiyichang pill group, the mucosal epithelium was intact at the ulcer-healing sites, and the large intestine glands were few. Glandular dilatation and a large number of inflammatory cells were observed at some sites (Fig. 1).

### Microbial diversity analysis

The results from the observed species index and the Shannon index showed there to be no significant difference in alpha diversity between the Garidisan group and model group (*P* = 0.82; *P* = 0.35). There was a significant difference in alpha diversity between the control group and the model group (*P* = 0.007; *P* = 0.02).

**Fig. 2:**
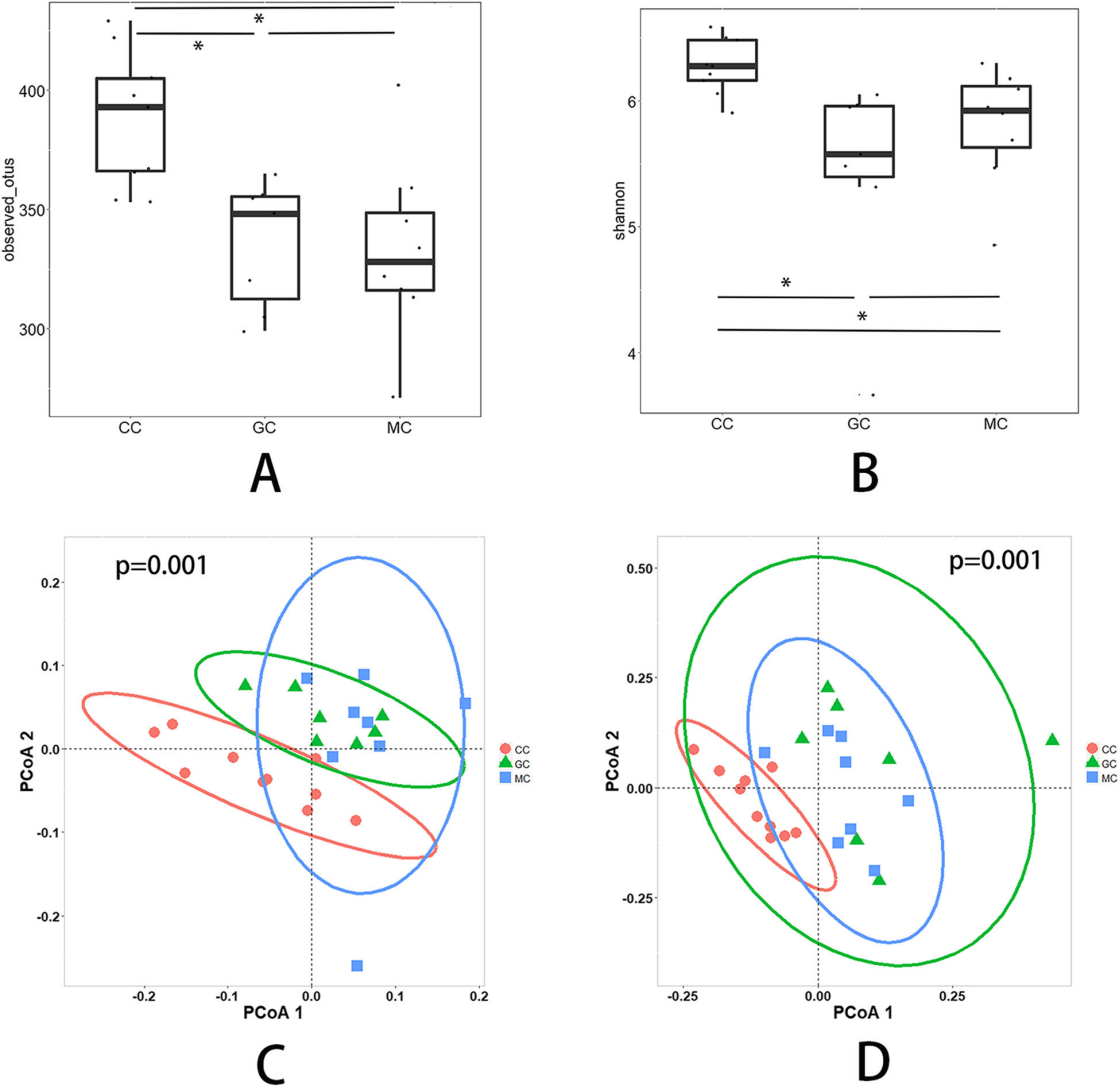
Analysis of bacterial community diversity. **a** Alpha diversity index (observed_species index) of intestinal contents within groups; **b** Alpha diversity index (Shannon index) of intestinal contents within groups; **c** Beta diversity index (Unifrac distance unweighted PCoA analysis) of intestinal contents between groups; **d** Beta diversity index (Unifrac distance weighted PCoA analysis) of intestinal contents between groups. *CC: the luminal content of the control group. MC: the luminal content of the model group. GC: the luminal content of the Garidisan group

Based on PCoA analysis of unweighted and weighted Unifrac distance, there was no overlap between the control group and the other two groups, indicating that there were few OTUs shared between the control group and the other two groups, and the number of differential OTUs was high (Fig. 2). Based on anosim analysis of weighted Unifrac distance, there was no significant difference in microbial community structure between the Garidisan group and the model group (*P* > 0.05). The community structure between the Garidisan group and the control group was significantly different, and the difference between the two groups was greater than the difference within each group (R > 0, *P* < 0.05) (Table 3). Based on the adnois analysis of weighted Unifrac distance, the grouping in this experiment demonstrated a high degree of interpretation of differences (Table 4).

**Table 3:** Weighted_unifrac anosim analysis

Compare	R_value	*P*_value
CC-GC	0.571861472	0.001
CC-MC	0.602739726	0.001
GC-MC	0.010932945	0.352

*CC* Luminal content of the control group. *MC* luminal content of the model group. *GC* Luminal content of the Garidisan group

**Table 4:** Weighted_unifrac adonis analysis

Compare	F_value	R2	*P*_value
CC-GC	6.683682522	0.308235583	0.001
CC-MC	7.167378836	0.309373749	0.001
GC-MC	1.039527854	0.074042935	0.36

*CC* Luminal content of the control group. *MC* Luminal content of the model group. *GC* Luminal content of the Garidisan group

### Differential microbiota analysis

**Fig. 3:**
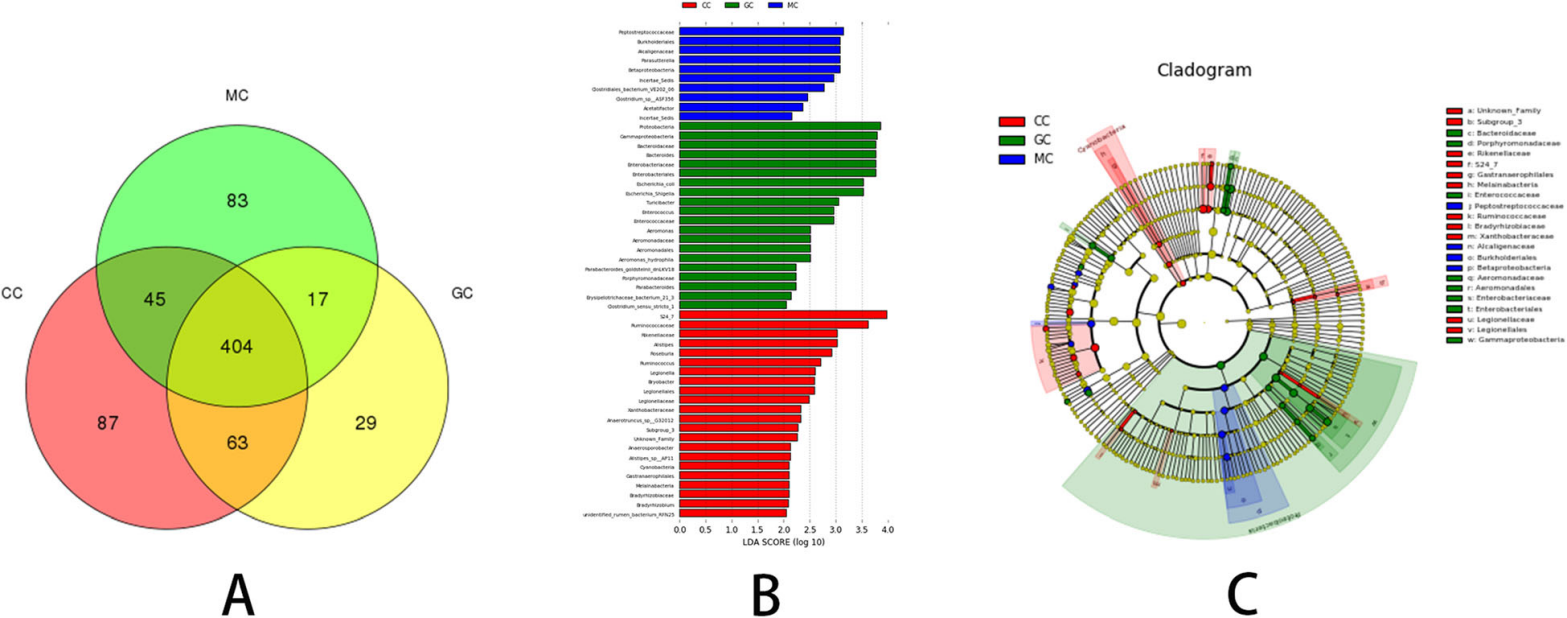
Analysis of the differential microbial community among the groups. **a** Venn diagram of differential microbial communities among the control group, model group, and Garidisan-treated group. **b** Histogram of LDA value distribution of the differential microbial community among the control group, model group, and Garidisan group; **c** Cladogram of LDA values from the control group, the model group, and Garidisan group. *CC: the luminal content of the control group. MC: the luminal content of the model group. GC: the luminal content of the Garidisan group

The number of OTUs shared by the groups was 404. The number of unique OTUs in the control group, model group, and Garidisan group were 87, 83, and 29, respectively (Fig. 3a). Metastatistical analysis showed that, at the phylum level, there were 16 phyla, including Firmicutes, Bacteroidetes, and Proteobacteria, that demonstrated significant differences in the relative abundance between the control group and the model group (Additional file 1: Table S1). There were 6 phyla, including Verrucomicrobia, Cyanobacteria, and Candidate_division_TM7, that demonstrated significant differences in the relative abundance between the Garidisan group and the model group (Additional file 2: Table S2). Comparison of the control group, model group, and Garidisan group showed that the biomarkers of the Garidisan group were Proteobacteria, Gammaproteobacteria, Bacteroidaceae, and others, for a total of 20 phyla (Fig. 3b, c).

### Regulatory effect of Garidisan on dysbiosis of the gut microbiota

A total of 141 differential OTUs related to UC were selected by the *P* value calculated by the rank sum test and the fold change in expression between two groups. There were 44 OTUs with a 2-folder or higher abundance in the model group than in the control group. There were 97 OTUs with a 0.5-fold or lower abundance in the model group than that in the control group (Additional file 3: Table S3).

**Fig. 4:**
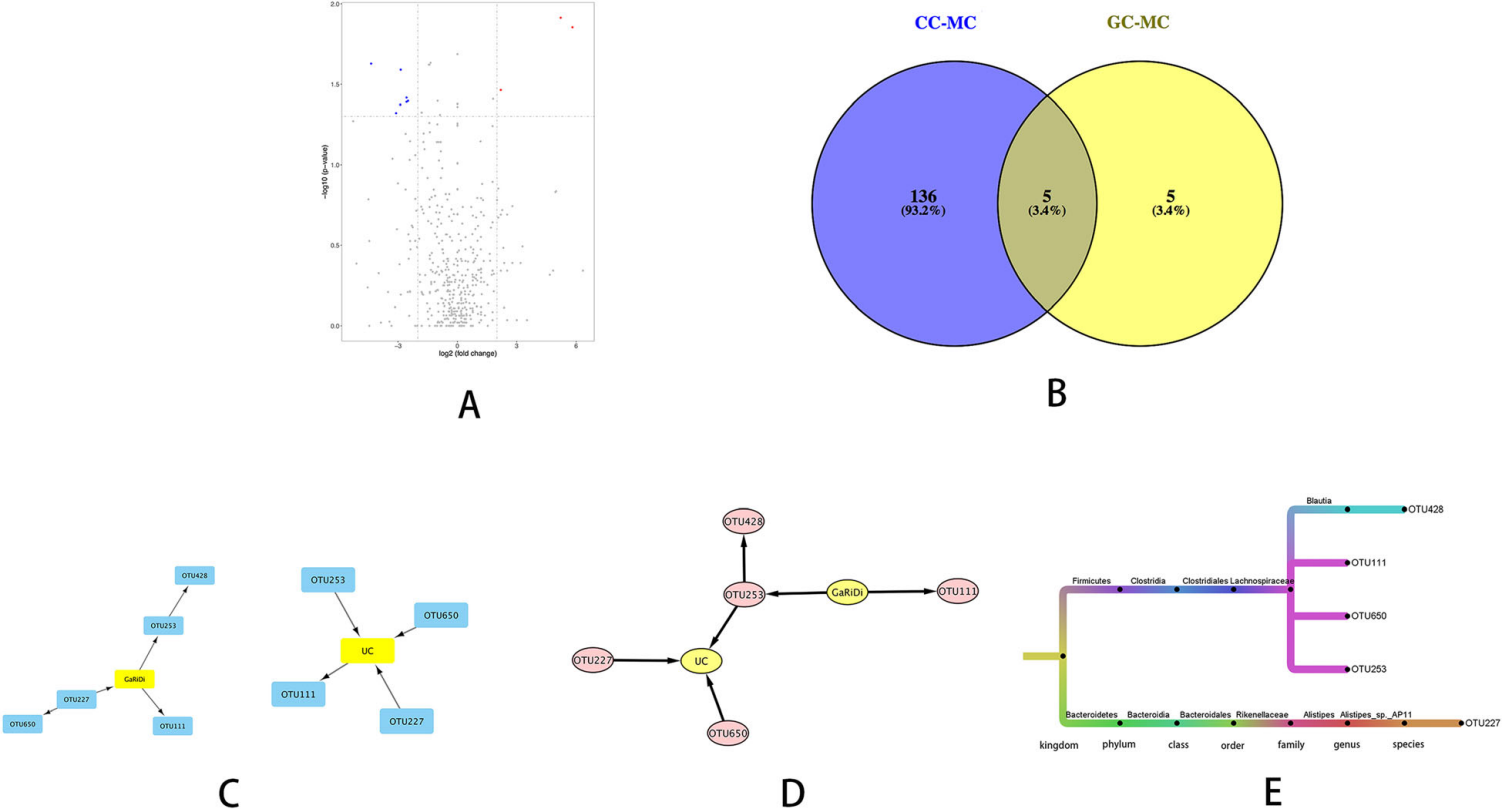
Garidisan regulated differential microbial communities related to UC. **a** Volcano plot of UC-related differential microbial communities between the Garidisan group and model group; **b** Venn diagram of identical differential OTUs in the Garidisan group (left circle is the differential OUTs between the control group and model group; right circle is the differential OTUs between the Garidisan group and model group). **c** Bayesian network analysis of the causal relationship of differential OTUs of the luminal contents to Garidisan and UC (left plot is the Bayesian network analysis of the causal relationship of differential OTUs of luminal content to Garidisan; the right plot is the Bayesian network analysis of the causal relationship of differential OTUs of luminal contents to UC); **d** Network diagram of mutual interactions between Garidisan, OUTs, and UC; **e** Species annotation of OTU428, OTU111, OTU650, OTU253, and OTU227

A total of 10 differential OTUs related to the model group were selected by the *P* value calculated from the rank sum test and the fold change in expression between the Garidisan and model groups. There were 3 OTUs with a 2-fold or higher abundance in the Garidisan group than in the model group. There were 7 OTUs with a 0.5-fold or lower abundance in the Garidisan group than in the model group (Additional file 4: Table S4, Fig. 4a). The 10 differential OTUs between the Garidisan and model groups were compared to the 141 differential OTUs between the control and model groups. Five identical differential OTUs (Fig. 4b) were obtained. These were OTU111, OTU650, OTU253, OTU428, and OTU227. This indicates that Garidisan may achieve its therapeutic effect by regulating the relative abundance of OTU111, OTU650, OTU253, OTU428, and OTU227, and may regulate the intestinal microflora dysbiosis in the model group. Bayesian network analysis was used for the five identical differential OTUs to determine the causal relationships of the identical differential OTUs to Garidisan and UC (Fig. 4c). According to the causal relationships of differential OTUs to Garidisan and UC, Garidisan can directly affect OTU253 to exert the therapeutic effect on UC (Fig. 4d).

## Discussion

With the rapid development in molecular genetics, molecular biology, and immunology in recent years, the understanding of UC has advanced remarkably. It is currently accepted that the pathogenesis of UC is closely related to environmental factors, genetic factors, intestinal microbes, and immune factors [1, 12]. Healthy adults host 500 to 1000 species of intestinal bacteria, and the number of genes encoded is 5 to 100 times that of human genes [13]. The composition and quantity of the gut microbiota are unevenly distributed. From the jejunum to the colon, the number of bacteria ranges from 10^4^ to 10^7^ bacteria per gram of intestinal contents in the jejunum to 10^11^ to 10^12^ bacteria per gram intestinal contents in the colon. They are mainly anaerobic bacteria and some facultative anaerobic bacteria. *Bacteroides*, *Prevotella*, and *Ruminococcus* bacteria in the gut microbiota are generally considered the dominant bacteria [14]. The key role of the dominant bacteria is to maintain intestinal microecological homeostasis. Nearly half of human feces is made up of bacteria. Human digestive bacteria include physiological bacteria, opportunistic pathogens, and pathogenic bacteria. Among them, there are numerous types of physiological bacteria, most of which are anaerobic and participate in the formation of mucosal barriers, maintain intestinal hemostasis, and prevent the intestinal mucosa from intestinal microbiota and various toxins to trigger an immune response [15]. The gut microbiota can ferment food residues that are not absorbed by the small intestine and provide nutrients and energy to the host. Results have shown there to be significant dysbiosis in the gut microbiota in UC patients. This is mainly reflected in the changes in the overall microbiota structure and bacterial abundance of the mucosa, a decrease in microbial diversity, and the alterations in the composition of the microbiota during the early stage of the disease, specifically, reduced probiotics and increased opportunistic pathogens [16–19].

Actinobacteria, Firmicutes, Bacteroidetes, Fusobacteria, and Proteobacteria are bacteria common in the human intestine [20]. The dominant bacterial genera reported in this study were *Bacteroides*, *Lactobacillus*, and *Blautia*, which are consistent with the literature. The type, quantity, and ratio of the gut microbials are related to genetic factors, diet, lifestyle, drugs, and host immune status. The gut microbiota can ferment food residues that are not absorbed by the small intestine, providing nutrients and energy to the host. Short-chain fatty acids such as acetic acid, propionic acid, and butyric acid produced by bacterial fermentation reduce the pH value and redox potential of the intestine to inhibit the growth of pathogenic bacteria and exert their bio-antagonistic effect. The gut microbiota is also involved in vitamin synthesis, as well as cancer prevention due to the decomposition of toxins and carcinogens in food [21, 22]. Many studies have shown there to be dysbiosis of the gut microbiota in UC patients. Dysbiosis is mainly manifested by the decreased diversity of the gut microbiota, lower quantity of probiotics as the dominant bacteria, and an increase in putrefying bacteria and pathogenic bacteria [13, 23]. This study showed the luminal microbiota of the model mice to be dysregulated. After screening, we found there to be 141 differential OTUs in the model group. When we used the model group as the reference, we found there to be 10 differential OTUs in the Garidisan group. The results indicated that when the model mice were treated with Garidisan, dysbiosis of the gut microbiota demonstrated different degrees of adjustment, suggesting the correlation between UC and dysbiosis of the gut microbiota.

Bacteria of the Lachnospiraceae family are anaerobic bacteria of the class Clostridia from the phylum Firmicutes [24]. Previous studies showed Lachnospiraceae in the intestine to participate in the metabolism of the host and interact with the microbes in the intestine. In addition, Lachnospiraceae is associated with diseases such as obesity, irritable bowel syndrome, and inflammatory bowel disease [24–27]. Lachnospiraceae is an important microbiome in the intestinal tract of ruminants and mammals, and is also important butyric acid-producing bacteria [28–30]. Butyric acid is usually produced by physiological bacteria in the intestine. It is a short-chain fatty acid found in the intestine and the best oxidative substrate for colonic epithelium. Its oxygen consumption can reach 80% of the oxygen consumption level of colon cells. Butyric acid can also reduce the inflammatory response by inhibiting the expression of cyclooxygenase and peroxidase and protect the intestinal mucosal barrier by inhibiting histone deacetylation and nuclear factor NF-κB activation [31, 32]. Butyric acid can provide important energy for the growth of microorganisms and promote the proliferation of host epithelial cells. Butyric acid can also be absorbed and utilized by colonic epithelial cells, and it is the preferred source of energy for the colon and cecum. Some studies reported that Lachnospiraceae showed an anti-colon cancer effect [33, 34]. In this study, the rank sum test was used to identify the shared differential bacteria, and the Bayesian causality network analysis was used to analyze the shared different bacteria. It was found that Garidisan can regulate dysbiosis of the gut microbiota in the intestinal tract and directly affect Lachnospiraceae for its therapeutic effect to UC.

UC is a chronic, spontaneous inflammatory bowel disease whose etiology is still unclear [35]. The mechanism by which Garidisan treats UC is still not clear, though previous studies have confirmed that Garidisan can regulate immune cells, balance pro-inflammatory factors and anti-inflammatory factors, and reduce the expression of ICAM-1 to lower the infiltration of inflammatory cells to promote tissue maturation of regenerative tissues. In addition, Garidisan can promote the functional maturity of the regenerative tissue by reducing collagen formation, promoting the maturation of new blood vessels, and increasing the expression of growth factors and their receptors. Garidisan can also repair the colonic mucosal epithelium of model rats [9]. This study was based on the current research progress of UC and the previous findings of Garidisan. Regarding the correlation between UC and dysbiosis of the gut microbiota, we here found that the mechanism by which Garidisan acts in the treatment of UC not only involves its regulation of cellular factors, such as immune factors, but also involves its regulatory effect on dysbiosis of the gut microbiota. Lachnospiraceae is an important target for studying the pathogenesis of UC and the regulatory effect of Garidisan on dysbiosis of the gut microbiota. More experiments are needed to validate these results.

## Conclusions

Garidisan has a good regulatory effect on the gut microbiota of the lumen. Lachnospiraceae could be an important and direct target for the study of the therapeutic mechanism by which Garidisan affects UC.

## Supplementary information

**Additional file 1:** **Table S1.** CC-MC (Phylum level) Metastat analysis.

**Additional file 2:** **Table S2.** GC-MC (Phylum level) Metastat analysis.

**Additional file 3:** **Table S3.** UC related difference species annotation table.

**Additional file 4:** **Table S4.** UC related difference species annotation table of GaRiDi pulvis.

## Data Availability

The datasets used and analysed during the current study available from the corresponding author on reasonable request.

## Abbreviations

16SrRNA: 16S ribosomal RNA
ANOVA: Analysis of Variance
CC: The luminal content of the control group
DAI: Disease active index
DSS: Dextran sodium sulphate
GC: The luminal content of the Garidisan group
IBD: Inflammatory browel disease
LDA: Linear Discriminant Analysis
LefSe: LDA Effect Size
MC: The luminal content of the model group
MetaStat: An improved statistical method for analysis of metagenomic data
MUC2: Mucin2
NMDS: Non-metric multidimentional scaling
OTU: Operational taxonomic unit
PBS: Phosphate buffer saline
PCA: Principal component analysis
PCoA: Principal Co-ordinates analysis
PCR: Polymerase Chain Reaction
TLR: Toll-like receptor
UC: Ulcerative colitis
