# Supplementary material for: Regulatory effect of Garidisan on dysbiosis of the gut microbiota in the mouse model of ulcerative colitis induced by dextran sulfate sodium
Source: BMC Complement Altern Med. 2019 Nov 21;19:329. doi: 10.1186/s12906-019-2750-y (PMC6873523; doi:10.1186/s12906-019-2750-y)
Supplement: Supplementary file 1 — Additional file 1: Table S1. CC-MC (Phylum level) Metastat analysis. [file 12906_2019_2750_MOESM1_ESM.doc]

Table S1. CC-MC (Phylum level) Metastat analysis

| Taxa | mean.CC. | variance.CC. | standard.error.CC. | mean.MC. | variance.MC. | standard.error.MC. | p.value | q.value |  |
| --- | --- | --- | --- | --- | --- | --- | --- | --- | --- |
| K_Bacteria; p__Firmicutes | 0.61109289985553 | 0.0115556269372038 | 0.033993568416987 | 0.65813864777788 | 0.0201993173182295 | 0.0502485289812417 | 0.445554445554446 | -0.0215975907740121 | |
| k__Bacteria;p__Bacteroidetes | 0.34932211903521 | 0.0126467243714467 | 0.0355622332980463 | 0.270721716744225 | 0.0169408248551308 | 0.0460174217757943 | 0.190809190809191 | -0.0215975907740121 | |
| k__Bacteria;p__Proteobacteria | 0.00965441527414234 | 3.49234330601403e-06 | 0.000590960515264263 | 0.0546576136222973 | 0.00111874797243347 | 0.0118255442392384 | 0.000999000999000999 | -0.0215975907740121 | |
| k__Bacteria;p__Verrucomicrobia | 0.0190583267429669 | 0.000220293343718469 | 0.00469354177267518 | 0.00704927357352531 | 3.00411860836128e-05 | 0.00193782049231904 | 0.036963036963037 | -0.0215975907740121 | |
| k__Bacteria;p__Actinobacteria | 0.00444511053523138 | 2.44488651021574e-06 | 0.000494457936554339 | 0.00364220117483752 | 1.8617718560115e-06 | 0.000482412149516818 | 0.268731268731269 | -0.0215975907740121 | |
| k__Bacteria;p__Deferribacteres | 0.00180863427189341 | 2.2243498705994e-06 | 0.000471630137989442 | 0.00373585831731741 | 8.78920662386206e-06 | 0.00104816545830454 | 0.0979020979020979 | -0.0215975907740121 | |
| k__Bacteria;p__Cyanobacteria | 0.00277159550546393 | 5.6311205111842e-06 | 0.000750407923144752 | 0.00115408651194041 | 1.00243738625322e-06 | 0.000353984001448727 | 0.0719280719280719 | -0.0215975907740121 | |
| k__Bacteria;p__Tenericutes | 0.00102545351015589 | 7.94444172693795e-08 | 8.91315978031245e-05 | 0.000529880466819652 | 9.11052473402093e-08 | 0.00010671530310844 | 0.003996003996004 | -0.0215975907740121 | |
| k__Bacteria;p__Candidate_division_TM7 | 0.000337251649236012 | 1.59601061214574e-08 | 3.99501015286037e-05 | 0.000174640370958349 | 3.92238788858609e-08 | 7.00213171879293e-05 | 0.0579420579420579 | -0.0215975907740121 | |
| k__Bacteria;p__Acidobacteria | 0.00022914464060953 | 4.94486959827265e-08 | 7.03197667677635e-05 | 6.18858160116022e-05 | 5.30679974049458e-09 | 2.57555812895345e-05 | 0.043956043956044 | -0.0215975907740121 | |
| k__Bacteria;p__Chloroflexi | 0.000110683337968573 | 1.06564544306455e-08 | 3.26442252636595e-05 | 5.03556177037748e-05 | 1.31838303333994e-09 | 1.2837362625068e-05 | 0.111888111888112 | -0.0215975907740121 | |
| k__Bacteria;p__Gemmatimonadetes | 9.37843127398505e-05 | 7.713945972282e-09 | 2.77739913809341e-05 | 2.0734766642357e-05 | 3.4748151484275e-10 | 6.5905378654055e-06 | 0.015984015984016 | -0.0215975907740121 | |
| k__Bacteria;p__Fusobacteria | 2.98530384061005e-05 | 5.87112286645025e-09 | 2.42304000512791e-05 | 1.12013054773258e-05 | 3.18341283958109e-10 | 6.30814239651925e-06 | 0.727272727272727 | -0.0215975907740121 | |
| k__Bacteria;p__Nitrospirae | 2.07282904464084e-05 | 1.32811003727957e-09 | 1.15243656540374e-05 | 1.26241975942682e-05 | 7.38755101100367e-10 | 9.6095987240647e-06 | 0.405219688338766 | -0.0215975907740121 | |
| k__Bacteria;p__Spirochaetae | 0 | 0 | 0 | 1.18364486866277e-05 | 1.12081214008936e-09 | 1.18364486866277e-05 | 0.0208644894327208 | -0.0215975907740121 | |
| k__Bacteria;p__JL-ETNP-Z39 | 0 | 0 | 0 | 6.13768044780517e-06 | 3.01368970234959e-10 | 6.13768044780517e-06 | 0.212698785080477 | -0.0215975907740121 | |

*CC: luminal content of the control group. MC: luminal content of the model group.
