# Supplementary material for: Regulatory effect of Garidisan on dysbiosis of the gut microbiota in the mouse model of ulcerative colitis induced by dextran sulfate sodium
Source: BMC Complement Altern Med. 2019 Nov 21;19:329. doi: 10.1186/s12906-019-2750-y (PMC6873523; doi:10.1186/s12906-019-2750-y)
Supplement: Supplementary file 2 — Additional file 2: Table S2. GC-MC (Phylum level) Metastat analysis. [file 12906_2019_2750_MOESM2_ESM.docx]

Table S2. GC-MC (Phylum level) Metastat analysis

| Taxa | mean.GC. | variance.GC. | standard.error.GC. | mean.MC. | variance.MC. | standard.error.MC. | p.value | q.value |  |
| --- | --- | --- | --- | --- | --- | --- | --- | --- | --- |
| k__Bacteria;p__Verrucomicrobia | 0.016805400624053 | 0.000180267317093056 | 0.00507468953438894 | 0.00704927357352531 | 3.00411860836128e-05 | 0.00193782049231904 | 0.0763846153846154 | 0.0335095052335594 | |
| k__Bacteria;p__Cyanobacteria | 0.000417712531631047 | 1.07364905795134e-07 | 0.000123846048322178 | 0.00115408651194041 | 1.00243738625322e-06 | 0.000353984001448727 | 0.052 | 0.0335095052335594 | |
| k__Bacteria;p__Candidate_division_TM7 | 3.25084733757695e-05 | 1.36396237452938e-09 | 1.39589314702061e-05 | 0.000174640370958349 | 3.92238788858609e-08 | 7.00213171879293e-05 | 0.0495384615384615 | 0.0335095052335594 | |
| k__Bacteria;p__Chloroflexi | 9.05831931499698e-05 | 2.12843116430414e-09 | 1.74373620396146e-05 | 5.03556177037748e-05 | 1.31838303333994e-09 | 1.2837362625068e-05 | 0.0676153846153846 | 0.0335095052335594 | |
| k__Bacteria;p__Gemmatimonadetes | 5.23929948365306e-05 | 2.28669169794218e-09 | 1.80740212062277e-05 | 2.0734766642357e-05 | 3.4748151484275e-10 | 6.5905378654055e-06 | 0.103769230769231 | 0.0390197000950122 | |
| k__Bacteria;p__Spirochaetae | 0 | 0 | 0 | 1.18364486866277e-05 | 1.12081214008936e-09 | 1.18364486866277e-05 | 0.0681572237454657 | 0.0335095052335594 | |

*MC: luminal content of the model group. GC: luminal content of the Garidisan group.
