# Supplementary material for: Regulatory effect of Garidisan on dysbiosis of the gut microbiota in the mouse model of ulcerative colitis induced by dextran sulfate sodium
Source: BMC Complement Altern Med. 2019 Nov 21;19:329. doi: 10.1186/s12906-019-2750-y (PMC6873523; doi:10.1186/s12906-019-2750-y)
Supplement: Supplementary file 3 — Additional file 3: Table S3. UC related difference species annotation table. [file 12906_2019_2750_MOESM3_ESM.doc]

Table S3 . UC related difference species annotation table

| OTU ID | *p*-value | Model group expression | Species Annotation |
| --- | --- | --- | --- |
| OTU102 | 0.001371178 | - | k__Bacteria; p__Bacteroidetes; c__Bacteroidia; o__Bacteroidales; f__S24-7 |
| OTU105 | 0.011163643 | - | k__Bacteria; p__Firmicutes; c__Clostridia; o__Clostridiales; f__Lachnospiraceae; g__Blautia |
| OTU107 | 4.57E-05 | - | k__Bacteria; p__Bacteroidetes; c__Bacteroidia; o__Bacteroidales; f__S24-7 |
| OTU109 | 0.00044562 | - | k__Bacteria; p__Bacteroidetes; c__Bacteroidia; o__Bacteroidales; f__Rikenellaceae; g__Alistipes |
| OTU110 | 0.000725179 | - | k__Bacteria; p__Firmicutes; c__Clostridia; o__Clostridiales; f__Ruminococcaceae |
| OTU113 | 0.000433573 | - | k__Bacteria; p__Firmicutes; c__Clostridia; o__Clostridiales; f__Ruminococcaceae; g__Anaerotruncus |
| OTU128 | 0.007467346 | - | k__Bacteria; p__Firmicutes; c__Clostridia; o__Clostridiales; f__Lachnospiraceae; g__Blautia |
| OTU129 | 0.002162272 | - | k__Bacteria; p__Firmicutes; c__Clostridia; o__Clostridiales; f__Ruminococcaceae |
| OTU132 | 0.000620296 | - | k__Bacteria; p__Firmicutes; c__Clostridia; o__Clostridiales; f__Ruminococcaceae |
| OTU141 | 0.000868413 | - | k__Bacteria; p__Firmicutes; c__Clostridia; o__Clostridiales; f__Ruminococcaceae |
| OTU144 | 0.000604305 | - | k__Bacteria; p__Firmicutes; c__Clostridia; o__Clostridiales; f__Lachnospiraceae |
| OTU149 | 0.006615442 | - | k__Bacteria; p__Firmicutes; c__Clostridia; o__Clostridiales; f__Ruminococcaceae; g__Ruminococcus |
| OTU152 | 0.00044562 | - | k__Bacteria; p__Firmicutes; c__Clostridia; o__Clostridiales; f__Lachnospiraceae |
| OTU153 | 0.008556168 | - | k__Bacteria; p__Firmicutes; c__Clostridia; o__Clostridiales; f__Lachnospiraceae; g__Roseburia |
| OTU158 | 0.01610731 | - | k__Bacteria; p__Cyanobacteria; c__Melainabacteria; o__Gastranaerophilales |
| OTU159 | 0.049779917 | - | k__Bacteria; p__Firmicutes; c__Bacilli; o__Lactobacillales; f__Lactobacillaceae; g__Lactobacillus; s__mouse_gut_metagenome |
| OTU160 | 0.023142634 | - | k__Bacteria; p__Firmicutes; c__Clostridia; o__Clostridiales; f__Lachnospiraceae; g__Roseburia |
| OTU172 | 0.000846477 | - | k__Bacteria; p__Firmicutes; c__Clostridia; o__Clostridiales; f__Lachnospiraceae; g__Roseburia |
| OTU174 | 0.000608276 | - | k__Bacteria; p__Firmicutes; c__Clostridia; o__Clostridiales; f__Lachnospiraceae; g__Marvinbryantia |
| OTU187 | 0.001295406 | - | k__Bacteria; p__Firmicutes; c__Clostridia; o__Clostridiales; f__Lachnospiraceae |
| OTU188 | 0.001573428 | - | k__Bacteria; p__Actinobacteria; c__Coriobacteriia; o__Coriobacteriales; f__Coriobacteriaceae |
| OTU189 | 0.00282827 | - | k__Bacteria; p__Cyanobacteria; c__Melainabacteria; o__Gastranaerophilales |
| OTU190 | 0.000830793 | - | k__Bacteria; p__Firmicutes; c__Clostridia; o__Clostridiales; f__Ruminococcaceae; g__Anaerotruncus |
| OTU191 | 0.001154762 | - | k__Bacteria; p__Firmicutes; c__Clostridia; o__Clostridiales; f__Lachnospiraceae |
| OTU194 | 0.00066692 | - | k__Bacteria; p__Firmicutes; c__Clostridia; o__Clostridiales; f__Lachnospiraceae; g__Blautia |
| OTU200 | 0.000851748 | - | k__Bacteria; p__Firmicutes; c__Clostridia; o__Clostridiales; f__Ruminococcaceae |
| OTU205 | 0.000390448 | - | k__Bacteria; p__Firmicutes; c__Clostridia; o__Clostridiales; f__Ruminococcaceae; g__Anaerotruncus |
| OTU207 | 0.000851748 | - | k__Bacteria; p__Firmicutes; c__Clostridia; o__Clostridiales; f__Christensenellaceae |
| OTU208 | 0.009821466 | - | k__Bacteria; p__Firmicutes; c__Clostridia; o__Clostridiales; f__Lachnospiraceae |
| OTU213 | 0.001435967 | - | k__Bacteria; p__Firmicutes; c__Clostridia; o__Clostridiales; f__Lachnospiraceae; g__Roseburia |
| OTU215 | 0.000442586 | - | k__Bacteria; p__Firmicutes; c__Clostridia; o__Clostridiales; f__Lachnospiraceae |
| OTU216 | 0.005166452 | - | k__Bacteria; p__Firmicutes; c__Clostridia; o__Clostridiales; f__Ruminococcaceae; s__unidentified_rumen_bacterium_RFN25 |
| OTU224 | 0.002117626 | - | k__Bacteria; p__Firmicutes; c__Clostridia; o__Clostridiales; f__Lachnospiraceae |
| OTU227 | 0.000510795 | - | k__Bacteria; p__Bacteroidetes; c__Bacteroidia; o__Bacteroidales; f__Rikenellaceae; g__Alistipes; s__Alistipes_sp._AP11 |
| OTU228 | 0.000427637 | - | k__Bacteria; p__Firmicutes; c__Clostridia; o__Clostridiales; f__Lachnospiraceae |
| OTU23 | 0.000857043 | - | k__Bacteria; p__Firmicutes; c__Clostridia; o__Clostridiales; f__Ruminococcaceae |
| OTU230 | 0.002777307 | - | k__Bacteria; p__Tenericutes; c__Mollicutes; o__RF9 |
| OTU231 | 0.003593057 | - | k__Bacteria; p__Actinobacteria; c__Coriobacteriia; o__Coriobacteriales; f__Coriobacteriaceae; g__Enterorhabdus |
| OTU233 | 0.000421759 | - | k__Bacteria; p__Firmicutes; c__Clostridia; o__Clostridiales; f__Lachnospiraceae |
| OTU245 | 0.004508753 | - | k__Bacteria; p__Firmicutes; c__Clostridia; o__Clostridiales; f__Lachnospiraceae |
| OTU247 | 0.000519263 | - | k__Bacteria; p__Firmicutes; c__Clostridia; o__Clostridiales; f__Ruminococcaceae |
| OTU249 | 0.002162272 | - | k__Bacteria; p__Firmicutes; c__Clostridia; o__Clostridiales; f__Ruminococcaceae |
| OTU250 | 0.001298732 | - | k__Bacteria; p__Firmicutes; c__Clostridia; o__Clostridiales; f__Clostridiaceae_1; g__Candidatus_Arthromitus |
| OTU253 | 0.005082761 | - | k__Bacteria; p__Firmicutes; c__Clostridia; o__Clostridiales; f__Lachnospiraceae |
| OTU259 | 0.001929936 | - | k__Bacteria; p__Firmicutes; c__Clostridia; o__Clostridiales; f__Ruminococcaceae; s__unidentified_rumen_bacterium_RFN71 |
| OTU263 | 0.001830907 | - | k__Bacteria; p__Firmicutes; c__Clostridia; o__Clostridiales; f__Ruminococcaceae |
| OTU265 | 0.001685009 | - | k__Bacteria; p__Actinobacteria; c__Coriobacteriia; o__Coriobacteriales; f__Coriobacteriaceae; g__Enterorhabdus |
| OTU266 | 0.006226015 | - | k__Bacteria; p__Tenericutes; c__Mollicutes; o__RF9 |
| OTU268 | 0.004288148 | - | k__Bacteria; p__Firmicutes; c__Clostridia; o__Clostridiales; f__Defluviitaleaceae; g__Incertae_Sedis |
| OTU27 | 0.005082761 | - | k__Bacteria; p__Firmicutes; c__Clostridia; o__Clostridiales; f__Ruminococcaceae |
| OTU273 | 0.00041032 | - | k__Bacteria; p__Firmicutes; c__Clostridia; o__Clostridiales; f__Peptococcaceae |
| OTU276 | 0.000393224 | - | k__Bacteria; p__Firmicutes; c__Clostridia; o__Clostridiales; f__Ruminococcaceae; g__Anaerotruncus |
| OTU285 | 0.025876838 | - | k__Bacteria; p__Firmicutes; c__Clostridia; o__Clostridiales; f__vadinBB60 |
| OTU287 | 0.000427637 | - | k__Bacteria; p__Firmicutes; c__Clostridia; o__Clostridiales; f__Ruminococcaceae |
| OTU288 | 0.009155193 | - | k__Bacteria; p__Firmicutes; c__Clostridia; o__Clostridiales; f__Defluviitaleaceae; g__Incertae_Sedis |
| OTU296 | 0.018987475 | - | k__Bacteria; p__Firmicutes; c__Clostridia; o__Clostridiales; f__Defluviitaleaceae; g__Incertae_Sedis |
| OTU297 | 0.003311597 | - | k__Bacteria; p__Firmicutes; c__Clostridia; o__Clostridiales; f__Family_XIII |
| OTU30 | 9.14E-05 | - | k__Bacteria; p__Firmicutes; c__Clostridia; o__Clostridiales; f__Lachnospiraceae; g__Blautia |
| OTU309 | 0.031281539 | - | k__Bacteria; p__Firmicutes; c__Clostridia; o__Clostridiales; f__Lachnospiraceae; s__Lachnospiraceae_bacterium_6-1 |
| OTU31 | 4.57E-05 | - | k__Bacteria; p__Bacteroidetes; c__Bacteroidia; o__Bacteroidales; f__S24-7 |
| OTU317 | 0.00206998 | - | k__Bacteria; p__Firmicutes; c__Clostridia; o__Clostridiales; f__Lachnospiraceae; g__Roseburia |
| OTU322 | 0.001514615 | - | k__Bacteria; p__Firmicutes; c__Clostridia; o__Clostridiales; f__Lachnospiraceae |
| OTU328 | 0.021821988 | - | k__Bacteria; p__Tenericutes; c__Mollicutes; o__RF9 |
| OTU33 | 0.000182824 | - | k__Bacteria; p__Firmicutes; c__Clostridia; o__Clostridiales; f__Lachnospiraceae; g__Incertae_Sedis |
| OTU331 | 0.001994628 | - | k__Bacteria; p__Firmicutes; c__Clostridia; o__Clostridiales; f__Lachnospiraceae |
| OTU338 | 0.000439567 | - | k__Bacteria; p__Firmicutes; c__Clostridia; o__Clostridiales; f__Lachnospiraceae; g__Roseburia |
| OTU340 | 0.021724432 | - | k__Bacteria; p__Firmicutes; c__Clostridia; o__Clostridiales; f__Ruminococcaceae; g__Anaerotruncus |
| OTU346 | 0.014374637 | - | k__Bacteria; p__Firmicutes; c__Clostridia; o__Clostridiales; f__Ruminococcaceae |
| OTU367 | 0.002093719 | - | k__Bacteria; p__Firmicutes; c__Clostridia; o__Clostridiales; f__Lachnospiraceae |
| OTU377 | 0.006185572 | - | k__Bacteria; p__Proteobacteria; c__Alphaproteobacteria; o__Sphingomonadales |
| OTU379 | 0.049219325 | - | k__Bacteria; p__Firmicutes; c__Clostridia; o__Clostridiales; f__Ruminococcaceae |
| OTU38 | 0.009936013 | - | k__Bacteria; p__Firmicutes; c__Clostridia; o__Clostridiales; f__Lachnospiraceae |
| OTU408 | 0.037642162 | - | k__Bacteria; p__Firmicutes; c__Clostridia; o__Clostridiales; f__Lachnospiraceae; g__Blautia |
| OTU41 | 0.011289781 | - | k__Bacteria; p__Firmicutes; c__Clostridia; o__Clostridiales; f__Ruminococcaceae; g__Ruminococcus |
| OTU418 | 0.047333461 | - | k__Bacteria; p__Gemmatimonadetes; c__Gemmatimonadetes; o__Gemmatimonadales; f__Gemmatimonadaceae |
| OTU42 | 0.000857043 | - | k__Bacteria; p__Firmicutes; c__Clostridia; o__Clostridiales; f__Lachnospiraceae |
| OTU432 | 0.000979794 | - | k__Bacteria; p__Firmicutes; c__Clostridia; o__Clostridiales; f__Lachnospiraceae |
| OTU433 | 0.049219325 | - | k__Bacteria; p__Firmicutes; c__Clostridia; o__Clostridiales; f__vadinBB60 |
| OTU48 | 0.000868413 | - | k__Bacteria; p__Firmicutes; c__Clostridia; o__Clostridiales; f__Lachnospiraceae; g__Roseburia |
| OTU513 | 0.041309694 | - | k__Bacteria; p__Acidobacteria; c__Acidobacteria; o__Subgroup_4; f__RB41 |
| OTU53 | 4.57E-05 | - | k__Bacteria; p__Bacteroidetes; c__Bacteroidia; o__Bacteroidales; f__S24-7 |
| OTU54 | 0.004387769 | - | k__Bacteria; p__Firmicutes; c__Clostridia; o__Clostridiales; f__Ruminococcaceae |
| OTU55 | 0.014446517 | - | k__Bacteria; p__Firmicutes; c__Erysipelotrichia; o__Erysipelotrichales; f__Erysipelotrichaceae; g__Allobaculum |
| OTU576 | 0.000387686 | - | k__Bacteria; p__Firmicutes; c__Clostridia; o__Clostridiales; f__Lachnospiraceae |
| OTU64 | 0.00044562 | - | k__Bacteria; p__Firmicutes; c__Clostridia; o__Clostridiales; f__Ruminococcaceae |
| OTU65 | 0.001175394 | - | k__Bacteria; p__Firmicutes; c__Clostridia; o__Clostridiales; f__Ruminococcaceae |
| OTU650 | 0.010665841 | - | k__Bacteria; p__Firmicutes; c__Clostridia; o__Clostridiales; f__Lachnospiraceae |
| OTU67 | 0.001371178 | - | k__Bacteria; p__Firmicutes; c__Clostridia; o__Clostridiales; f__Lachnospiraceae |
| OTU69 | 0.034279446 | - | k__Bacteria; p__Bacteroidetes; c__Bacteroidia; o__Bacteroidales; f__S24-7 |
| OTU77 | 0.000319941 | - | k__Bacteria; p__Firmicutes; c__Clostridia; o__Clostridiales; f__Ruminococcaceae; g__Anaerotruncus; s__Anaerotruncus_sp._G32012 |
| OTU81 | 9.14E-05 | - | k__Bacteria; p__Firmicutes; c__Clostridia; o__Clostridiales; f__Defluviitaleaceae |
| OTU813 | 9.14E-05 | - | k__Bacteria; p__Firmicutes; c__Clostridia; o__Clostridiales; f__Lachnospiraceae; g__Incertae_Sedis |
| OTU85 | 0.00044562 | - | k__Bacteria; p__Firmicutes; c__Clostridia; o__Clostridiales; f__Lachnospiraceae |
| OTU87 | 0.004387769 | - | k__Bacteria; p__Firmicutes; c__Clostridia; o__Clostridiales; f__Ruminococcaceae |
| OTU89 | 0.00044562 | - | k__Bacteria; p__Bacteroidetes; c__Bacteroidia; o__Bacteroidales; f__S24-7 |
| OTU896 | 0.005037231 | - | k__Bacteria; p__Firmicutes; c__Clostridia; o__Clostridiales; f__Ruminococcaceae |
| OTU99 | 0.000433573 | - | k__Bacteria; p__Firmicutes; c__Clostridia; o__Clostridiales; f__Lachnospiraceae |
| OTU103 | 0.045366123 | + | k__Bacteria; p__Firmicutes; c__Clostridia; o__Clostridiales; f__Lachnospiraceae; g__Acetatifactor |
| OTU111 | 0.008728795 | + | k__Bacteria; p__Firmicutes; c__Clostridia; o__Clostridiales; f__Lachnospiraceae |
| OTU124 | 0.00116849 | + | k__Bacteria; p__Firmicutes; c__Clostridia; o__Clostridiales; f__Ruminococcaceae; g__Anaerotruncus |
| OTU125 | 0.043369513 | + | k__Bacteria; p__Firmicutes; c__Clostridia; o__Clostridiales; f__Lachnospiraceae; g__Acetatifactor |
| OTU127 | 0.000436563 | + | k__Bacteria; p__Firmicutes; c__Clostridia; o__Clostridiales; f__Lachnospiraceae |
| OTU145 | 0.000986521 | + | k__Bacteria; p__Firmicutes; c__Clostridia; o__Clostridiales; f__Clostridiaceae_1; g__Clostridium_sensu_stricto_1 |
| OTU15 | 4.57E-05 | + | k__Bacteria; p__Firmicutes; c__Clostridia; o__Clostridiales; f__Peptostreptococcaceae |
| OTU150 | 0.001478052 | + | k__Bacteria; p__Firmicutes; c__Erysipelotrichia; o__Erysipelotrichales; f__Erysipelotrichaceae; g__Holdemania; s__Holdemania_filiformis_DSM_12042 |
| OTU162 | 0.005906156 | + | k__Bacteria; p__Firmicutes; c__Clostridia; o__Clostridiales; f__Ruminococcaceae; g__Flavonifractor |
| OTU18 | 4.57E-05 | + | k__Bacteria; p__Proteobacteria; c__Betaproteobacteria; o__Burkholderiales; f__Alcaligenaceae; g__Parasutterella |
| OTU180 | 0.031354665 | + | k__Bacteria; p__Firmicutes; c__Clostridia; o__Clostridiales; f__Ruminococcaceae; g__Incertae_Sedis; s__bacterium_NLAE-zl-H60 |
| OTU192 | 0.040784332 | + | k__Bacteria; p__Firmicutes; c__Clostridia; o__Clostridiales; f__Lachnospiraceae |
| OTU197 | 0.020599541 | + | k__Bacteria; p__Firmicutes; c__Clostridia; o__Clostridiales; f__Ruminococcaceae; g__Anaerotruncus |
| OTU198 | 0.003687216 | + | k__Bacteria; p__Firmicutes; c__Clostridia; o__Clostridiales; f__Ruminococcaceae |
| OTU2 | 0.00044562 | + | k__Bacteria; p__Bacteroidetes; c__Bacteroidia; o__Bacteroidales; f__Bacteroidaceae; g__Bacteroides |
| OTU202 | 0.000407314 | + | k__Bacteria; p__Firmicutes; c__Clostridia; o__Clostridiales; f__Lachnospiraceae |
| OTU220 | 0.004182758 | + | k__Bacteria; p__Firmicutes; c__Clostridia; o__Clostridiales; f__Ruminococcaceae |
| OTU234 | 0.04845523 | + | k__Bacteria; p__Proteobacteria; c__Gammaproteobacteria; o__Enterobacteriales; f__Enterobacteriaceae; g__Proteus; s__Proteus_mirabilis |
| OTU237 | 0.000433573 | + | k__Bacteria; p__Firmicutes; c__Clostridia; o__Clostridiales; f__Peptococcaceae; g__Peptococcus |
| OTU24 | 0.020521962 | + | k__Bacteria; p__Firmicutes; c__Clostridia; o__Clostridiales; f__Lachnospiraceae; g__Blautia |
| OTU243 | 0.019745953 | + | k__Bacteria; p__Cyanobacteria; c__Melainabacteria; o__Gastranaerophilales |
| OTU248 | 0.002865826 | + | k__Bacteria; p__Firmicutes; c__Clostridia; o__Clostridiales; f__vadinBB60 |
| OTU25 | 0.000442586 | + | k__Bacteria; p__Firmicutes; c__Clostridia; o__Clostridiales; f__Lachnospiraceae; g__Incertae_Sedis |
| OTU280 | 0.019000284 | + | k__Bacteria; p__Firmicutes; c__Clostridia; o__Clostridiales; f__Lachnospiraceae |
| OTU29 | 0.014497231 | + | k__Bacteria; p__Firmicutes; c__Clostridia; o__Clostridiales; f__Lachnospiraceae |
| OTU3 | 4.57E-05 | + | k__Bacteria; p__Proteobacteria; c__Gammaproteobacteria; o__Enterobacteriales; f__Enterobacteriaceae; g__Escherichia-Shigella; s__Escherichia_coli |
| OTU32 | 0.004424253 | + | k__Bacteria; p__Firmicutes; c__Clostridia; o__Clostridiales; f__Lachnospiraceae |
| OTU344 | 0.006634712 | + | k__Bacteria; p__Firmicutes; c__Clostridia; o__Clostridiales; f__Christensenellaceae |
| OTU35 | 0.005014552 | + | k__Bacteria; p__Bacteroidetes; c__Bacteroidia; o__Bacteroidales; f__Porphyromonadaceae; g__Parabacteroides; s__Parabacteroides_goldsteinii_dnLKV18 |
| OTU39 | 0.00044562 | + | k__Bacteria; p__Firmicutes; c__Clostridia; o__Clostridiales; f__Lachnospiraceae; g__Blautia; s__Clostridiales_bacterium_VE202-06 |
| OTU428 | 0.01610731 | + | k__Bacteria; p__Firmicutes; c__Clostridia; o__Clostridiales; f__Lachnospiraceae; g__Blautia |
| OTU45 | 0.045477611 | + | k__Bacteria; p__Firmicutes; c__Clostridia; o__Clostridiales; f__Lachnospiraceae; g__Blautia |
| OTU505 | 0.000182824 | + | k__Bacteria; p__Bacteroidetes; c__Bacteroidia; o__Bacteroidales; f__Bacteroidaceae; g__Bacteroides |
| OTU56 | 0.004424253 | + | k__Bacteria; p__Firmicutes; c__Clostridia; o__Clostridiales; f__Lachnospiraceae |
| OTU57 | 0.001861789 | + | k__Bacteria; p__Firmicutes; c__Clostridia; o__Clostridiales; f__Defluviitaleaceae; s__Clostridium_sp._ASF356 |
| OTU6 | 0.000620296 | + | k__Bacteria; p__Bacteroidetes; c__Bacteroidia; o__Bacteroidales; f__Bacteroidaceae; g__Bacteroides |
| OTU60 | 0.000720578 | + | k__Bacteria; p__Firmicutes; c__Erysipelotrichia; o__Erysipelotrichales; f__Erysipelotrichaceae; g__Incertae_Sedis; s__Erysipelotrichaceae_bacterium_21_3 |
| OTU644 | 0.000715997 | + | k__Bacteria; p__Firmicutes; c__Clostridia; o__Clostridiales; f__Lachnospiraceae |
| OTU695 | 0.00562725 | + | k__Bacteria; p__Firmicutes; c__Clostridia; o__Clostridiales; f__Lachnospiraceae |
| OTU7 | 0.00044562 | + | k__Bacteria; p__Firmicutes; c__Erysipelotrichia; o__Erysipelotrichales; f__Erysipelotrichaceae; g__Turicibacter |
| OTU73 | 0.002493726 | + | k__Bacteria; p__Firmicutes; c__Clostridia; o__Clostridiales; f__Family_XIII |
| OTU75 | 0.034279446 | + | k__Bacteria; p__Firmicutes; c__Clostridia; o__Clostridiales; f__Lachnospiraceae |
| OTU9 | 0.000620296 | + | k__Bacteria; p__Firmicutes; c__Erysipelotrichia; o__Erysipelotrichales; f__Erysipelotrichaceae; g__Incertae_Sedis |
| OTU90 | 0.000857043 | + | k__Bacteria; p__Firmicutes; c__Clostridia; o__Clostridiales; f__Lachnospiraceae |

*“+”means that the OTU abundance in model group is higher than or equal to 2 times the control group;“-”means that the OTU abundance in model group is 1/2 or less than 1/2 of the control group.
