# Supplementary material for: Regulatory effect of Garidisan on dysbiosis of the gut microbiota in the mouse model of ulcerative colitis induced by dextran sulfate sodium
Source: BMC Complement Altern Med. 2019 Nov 21;19:329. doi: 10.1186/s12906-019-2750-y (PMC6873523; doi:10.1186/s12906-019-2750-y)
Supplement: Supplementary file 4 — Additional file 4: Table S4. UC related difference species annotation table of GaRiDi pulvis. [file 12906_2019_2750_MOESM4_ESM.docx]

Table S4. UC related difference species annotation table of GaRiDi pulvis group

| OTU ID | *p*-value | Model group expression | Species Annotation |
| --- | --- | --- | --- |
| OTU227 | 0.034347155 | + | k__Bacteria; p__Bacteroidetes; c__Bacteroidia; o__Bacteroidales; f__Rikenellaceae; g__Alistipes; s__Alistipes_sp._AP11 |
| OTU46 | 0.013986014 | + | k__Bacteria; p__Proteobacteria; c__Gammaproteobacteria; o__Enterobacteriales; f__Enterobacteriaceae |
| OTU650 | 0.012206879 | + | k__Bacteria; p__Firmicutes; c__Clostridia; o__Clostridiales; f__Lachnospiraceae |
| OTU111 | 0.04009324 | - | k__Bacteria; p__Firmicutes; c__Clostridia; o__Clostridiales; f__Lachnospiraceae |
| OTU201 | 0.047728227 | - | k__Bacteria; p__Firmicutes; c__Clostridia; o__Clostridiales; f__Lachnospiraceae |
| OTU223 | 0.025686161 | - | k__Bacteria; p__Bacteroidetes; c__Bacteroidia; o__Bacteroidales; f__S24-7 |
| OTU232 | 0.038264534 | - | k__Bacteria; p__Candidate_division_TM7; c__Unknown_Class; o__Unknown_Order; f__Unknown_Family; g__Candidatus_Saccharimonas |
| OTU253 | 0.040630065 | - | k__Bacteria; p__Firmicutes; c__Clostridia; o__Clostridiales; f__Lachnospiraceae |
| OTU428 | 0.04228766 | - | k__Bacteria; p__Firmicutes; c__Clostridia; o__Clostridiales; f__Lachnospiraceae; g__Blautia |
| OTU575 | 0.023562079 | - | k__Bacteria; p__Firmicutes; c__Clostridia; o__Clostridiales; f__Lachnospiraceae; g__Blautia |

*“+”means that the OTU abundance in GaRiDi pulvis group is higher than or equal to 2 times the model group;“-”means that the OTU abundance in GaRiDi pulvis group is 1/2 or less than 1/2 of the model group.
